# An overview of targeted cancer therapy

**DOI:** 10.7603/s40681-015-0019-4

**Published:** 2015-11-28

**Authors:** Viswanadha Vijaya Padma

**Affiliations:** 1 Department of Biotechnology, Bharathiar University, 641 046 Coimbatore, Tamil Nadu India; 2 Department of Health and Nutrition Biotechnology, Asia University, 413 Taichung, Taiwan

**Keywords:** Chemotherapy, Multidrug resistance, Targeted therapy, Prodrug, Small molecule inhibitors, Nano-particulate antibody conjugates

## Abstract

Cancer is a multifactorial disease and is one of the leading causes of death worldwide. The contributing factors include specific genetic background, chronic exposure to various environmental stresses and improper diet. All these risk factors lead to the accumulation of molecular changes or mutations in some important proteins in cells which contributes to the initiation of carcinogenesis. Chemotherapy is an effective treatment against cancer but undesirable chemotherapy reactions and the development of resistance to drugs which results in multi-drug resistance (MDR) are the major obstacles in cancer chemotherapy. Strategies which are in practice with limited success include alternative formulations e.g., liposomes, resistance modulation e.g., PSC833, antidotes/toxicity modifiers e.g., ICRF-187 and gene therapy. Targeted therapy is gaining importance due to its specificity towards cancer cells while sparing toxicity to off-target cells. The scope of this review involves the various strategies involved in targeted therapy like-monoclonal antibodies, prodrug, small molecule inhibitors and nano-particulate antibody conjugates.

## 1. Introduction

Cancer is the second leading cause of deaths all over the world. Globally 7.6 million deaths are caused by cancer which represents 13% of all global deaths [1]. Surgery, chemotherapy, and irradiation are the mainstream therapeutic approaches for cancer, chemotherapy being an important component of treatment for cancer patients. However, its success is limited due to lack of selectivity for tumor cells over normal cells resulting in insufficient drug concentrations in tumors, systemic toxicity and the appearance of drug-resistant tumor cells [2]. Several strategies have been proposed which include alternative formulations e.g., liposomes [3], resistance modulation e.g., PSC833 [4], antidotes/toxicity modifiers e.g., ICRF-187 [5] and gene therapy. Recently targeted therapy is gaining importance due to its specificity towards cancer cells while sparing toxicity to off-target cells.

Targeted therapy aims at delivering drugs to particular genes or proteins that are specific to cancer cells or the tissue environment that promotes cancer growth. Effectiveness of the therapy lies in targeted release of therapeutics at the disease site while minimizing the off-target side effects caused to normal tissues. It is often used in conjunction with chemotherapy and other cancer treatments. Targeted therapy involves developing drugs that block cancer cell proliferation, promote cell cycle regulation or induce apoptosis or autophagy and targeted delivery of toxic substances specifically to cancer cells to destroy them. Targeted therapy involves the use of monoclonal antibodies or oral small drugs [6].

Monoclonal antibodies are the focus of intense research in the field of cancer therapeutics since mid 1970s when the customized monoclonal antibody production was reported. Monoclonal antibody production, antibody engineering with display and screening innovations such as phage display meant the binding of antibody to a wide range of targeted antigens with exceptional specificity. Cancer immunotherapy involves the use of gemtuzumab (Mylotarg^®^; Wyeth, CT, USA), a CD-33 specific monoclonal antibody conjugated to a calicheamicin used for the treatment of acute myeloid leukemia [7]. On a similar note, radioisotope conjugated targeting antibodies have been developed for imaging (immunoscintigraphy) and radioimmunotherapy strategies. ^90^Y metal isotope based anti-CD20 ibritumomabtiuxetan (Zevalin^®^; Spectrum Pharmaceuticals, CA, USA) has been developed for use in clinical therapy [8, 9].

Moreover, apart from being used as therapeutic agents antibodies also serve as targeting agents. They are used in targeted therapy for the delivery of active therapeutics [10], prodrug activation enzymes [11, 12] and chemotherapy toxins [13-15]. Monoclonal antibodies block a specific target on the outside of cancer cells or in the tissue surrounding it. Monoclonal antibodies are used to deliver chemotherapeutic drugs and radioactive substances, directly to cancer cells. Being large compounds these drugs are usually given intravenously.

Prodrug cancer therapy involves selective activation of prodrug(s) in tumor tissues by exogenous enzyme(s) which can be accomplished by several methods which include: gene-directed enzyme prodrug therapy (GDEPT), virus-directed enzyme prodrugtherapy (VDEPT), and antibody-directed enzyme prodrug therapy (ADEPT). The important aspect of prodrug cancer therapy is to deliver drug-activating enzyme or gene or functional protein to tumor tissues, followed by systemic administration of a prodrug [2].

Prodrug cancer therapy is a two step process, the first step involves, targeting the drug-activating enzyme and its expression in tumors followed by the systemic administration of the nontoxic prodrug, which is the substrate for the exogenous enzyme that is targeted and expressed in tumors [16-18]. This in turn helps in localization of activated anticancer drug (toxic drug) in high concentration in tumors. The success of the prodrug therapy requires that both enzymes and prodrugs should meet certain criteria: the enzyme should be of nonhuman origin or a human protein either absent or has low expression levels in normal tissues [19, 20] but should find sufficient expression in tumors with high catalytic activity [21]. The prodrug should not be activated by the endogenous enzymes in non-tumor tissues but must be a good substrate for the expressed enzyme in tumors. The prodrug should be highly diffusible and be activated in the tumor cell with high cytotoxic potential. Further, it must exhibit ‘bystander’ killing effect by being actively taken up by the nonexpressing neighboring cancer cells. The half life of the prodrug should be long enough to exhibit bystander effect but should not permit drug leakage to systemic ciruculation [22]. The targeting strategies for enzyme/ prodrug can be divided into two major classes: (a) delivery of genes that encode prodrug-activating enzymes to tumor tissues (GDEPT, VDEPT, GPAT etc.); and (b) delivery of active enzymes onto tumor tissues (ADEPT).

Gene directed enzyme prodrug therapy, is a technique that involves delivery of a gene that encodes a foreign enzyme to tumor cells where it finds expression and activates a systemically administered nontoxic prodrug [16, 23, 24]. The enzyme/prodrug systems applied in GDEPT include: HSV-TK/GCV, *Escherichia coli* CD/5-FC and *E. coli* NTR/CB1954 which act intracellularly by converting prodrugsinto active drugs within cancer. Cell-cell contact is essential for this mode of action for effective killing. An extra-cellular cytotoxic effector system includes the conversion of an inactive glucuronidated derivative of doxorubicin (HMR 1826) to the cytotoxic doxorubicin in the tumor cells by the secreted form of lysosomal human glucuronidase. In the extracellular system the hydrophilic prodrug gets converted into a lipophilic, cell-permeable cytotoxic drug outside cells and hence targets both transduced and nontransduced cells. It exhibits enhanced cytotoxic potential as cell-cell contact is not required for a bystander effect [16].

Virus directed enzyme prodrug therapy (VDEPT) uses viral vectors to deliver a gene that encodes an enzyme that can convert a systemically administrated nontoxic prodrug into a cytotoxic agent within tumor cells. The NTR/CB1954 combination is used against colorectal and pancreatic cancer cells to sensitize them to CB1954 after retro-viral transduction and expression of the *E. coli* NTR gene [25, 26].

The viruses used for VDEPT include: retroviruses, adenoviruses, HSV [27], adeno-associated virus [28-30], lentivirus and EBV [31]. Over the years, many drug-activating enzyme gene/ prodrug combinations have been delivered into tumors *in vitro* or *in vivo* by VDEPT, the majority using CD/5-FC or HSV-TK/GCV with the involvement of retroviral and adenoviral vectors [32].

Genetic prodrug activation therapy (GPAT) induces the selective expression of a drug-metabolizing enzyme for activation of prodrug into a toxic moiety using the known transcriptional differences between normal and tumor cells [33, 34]. Several tumorspecific Transription responsive elements (TREs) have been used, which include genes that are either tumor specific or tumor associated antigens, such as CEA for colorectal cancer or N-myc for neuroblastoma [2].

Antibody directed enzyme prodrug therapy (ADEPT) uses a conjugate which consists of tumor specific antibody linked to a drug-activating enzyme which when administered systemically targets tumor tissues. This targeted enzyme which is localized on the tumor surface, converts the systemically administered nontoxic prodrug into a toxic drug resulting in cytotoxic effects in tumor cells [12, 35-40]. The ideal drugs for ADEPT include diffusible small molecules, which can diffuse in to both antigen-positive and antigen-negative tumor cells, and cause a bystander effect [35-37]. The interval between enzyme and prodrug administrations should be optimized to enhance the conjugate accumulation in tumors and avoid their leakage to blood and normal tissues, to avoid systemic toxicity.

The important criteria for ADEPT include: the target antigen should be accessible, therefore it should preferably be a membrane bound antigen associated with the tumor cell membrane or secreted into the extracellular matrix of the tumor [41], and the antibody should be a monoclonal antibody with high affinity [35]. The enzyme should have optimal activity at a pH close to that of the tumor extracellular fluid.

The interval between enzyme and prodrug administrations is important for ADEPT, studies carried out in animals regarding the optimal interval showed that with the enzyme CPG2 linked to the anti-CEA antibody A5B7, the prodrug CMDA can be safely given 48 h or 72 h after antibody-enzyme administration [36]. In human subjects, the prodrug can be administered safely after 7 days to avoid systemic toxicity due to the activation of prodrug in plasma, as it takes 7 days for the adequate clearance of antibodyenzyme conjugate from the plasma [35]. The Phase I clinical trials carried out with CMDA/CPG2 prodrug/ enzyme system in colorectal carcinoma patients has revealed promising results. The bacterial enzyme CPG2 was conjugated to the F(ab)2 fragment of murine A5B7 monoclonal Ab, and a galactosylated second clearing Ab against CPG2 was also used to lower levels of conjugate in the circulation and other nontumor tissues. The plasma levels of the prodrug CMDA and active drug CJS11, a bifunctional alkylating agent, released from prodrug by the action of CPG2 localized in tumors were measured. The results showed that after applying the clearing agent, CPG2 activity was found in metastatic tumor biopsies, but not found in normal tissues. Further, a rapid appearance of the active drug with half-life of 36 ± 14 min in plasma was encouraging [42].

The limitations of ADEPT include: restricted delivery of the large conjugate in poorly vascularized tumors, therefore it is not possible to deliver antibody/enzyme conjugate to all of the tumor cells [43]. With low levels of the enzyme, adequate quantities of active drug to reach the cytotoxic concentration cannot be achieved. The antigen heterogeneity does not permit the binding of the conjugate to the cell surface. Other disadvantages of ADEPT include cost and availability of purified antibodies, immunogenicity of antibodies, accessibility of tumor to the enzyme/ antibody conjugate, and the conversion of prodrugs in nontumor tissues [41]. The main problem being the immunogenicity of the antibody-enzyme conjugate, which limits multiple cycles of its application this can be overcome with the use of humanized proteins and concomitant administration of immunosuppression [35].

## 2. Small molecule inhibitors in cancer therapy

Small drugs constitute a pill that a patient takes orally. As they are smaller chemical components than monoclonal antibodies, the body absorbs them better. The small drugs usually are targeted against specific molecular targets which are important for cancer cell proliferation or metastasis or angiogenesis. The current state of cancer drug discovery and development focuses on small molecule inhibitors which act against new molecular targets that determine therapeutic outcome. The molecularly targeted cancer therapies have resulted in improving the lives of a large number of cancer patients. The successful treatment of patients with acute promyelocytic leukaemia harbouring translocations in the *RARα* retinoic acid receptor gene with all-*trans* retinoic acid [44] and chronic myeloid leukaemia in which the malignancy is driven by the *BCR-ABL* translocation with the ABL inhibitor imatinib [45, 46] serve as the proof of the concept of molecular targeting of pathogenetic driver abnormalities with a small molecule in the clinical setting. The other small molecule inhibitors of cancer targets include, e.g. the gefitinib - inhibitor of epidermal growth factor receptor (EGFR) kinase and erlotinib- the inhibitor of EGFR in non small cell lung cancer (NSCLC) patients; the lapatinib- inhibitor of EGFR/ERBB2 for ERBB2-positive breast cancer; and the sorafenib- inhibitor of vascular epidermal growth factor receptor (VEGFR) kinase, in renal cancer [47]. The recent addition to the list is, abiraterone- the CYP171A1 inhibitor which blocks androgen synthesis, approved for treatment of late stage, castrationresistant prostate cancer [48], crizotinib -inhibitor of the protein kinase ALK approved for the treatment of NSCLC patients with a pathogenic rearrangement of the *ALK* gene [49] and vemurafenib – inhibitor of *BRAF* kinase [50] for metastatic melanoma with the BRAF V600E mutation. The progress with small molecule drugs is mirrored by the successful introduction of protein-based therapeutics, particularly antibodies, as exemplified by the anti- ERBB2 monoclonal antibody trastuzumab in ERBB2-positive breast cancer [51, 52]. These examples provide ample evidence of the success in targeting the pathogenic drivers to which cancer cells are ‘addicted’ [53, 54].

Despite the considerable progress in cancer therapy with the advent of new molecularly targeted therapies, therapeutic options are still limited for many patients and the process of new drug development is frustratingly slow with high failure rates [52, 55], The reasons for slow progress is that, frequently, a patient with a particular anatomically and histologically defined solid tumor respond to the treatment with a particular class of kinase inhibitor that matches the predominant pathogenic driver mutation e.g., NSCLC patients with EGFR mutations respond to EGFR inhibitors while those with ALK translocations respond to ALK inhibitors [47, 56]. Which necessitates understanding the value of specific gene targeting and selection of patient specific companion biomarkers on cancer drug discovery.

Another important task is identification of specific molecular targets through the sequencing of various cancer genomes which revealed extraordinary complexity with several genetic alterations and considerable genetic heterogeneity, not only between different tumours but also within an individual cancer [57-59]. Further, the heterogeneous population of tumors also include drug-resistant stem [60] and other host cells which aid in tumour progression [47]. This heterogeneity leads to drug resistance and the need for combinatorial therapy.

After identification of a potential novel therapeutic target, the next challenge is that of selecting and validating the best targets. This requires establishing a causal linkage of the proposed target and target modulation to deliver a therapeutically meaningful biological effect in relevant experimental models. ‘Druggability gap’ is the main concern in the drug discovery for medicinal chemists using small molecules. Frequently, the promising targets are regarded as technically undruggable as they cannot be targeted with small molecules which is referred to as ‘druggability gap’ e.g., RAS proteins, c-MYC or hypoxia inducible factor (HIF) [61]. Unfortunately, sometimes, cancer cells develop resistance for drugs with therapeutic efficacy as proved by the successful completion of clinical trials as shown recently by crizotinib [62] and vemurafenib [63]. This could be due to the mutation of the target gene [64], or activation of feedback loops [65] or development of alternative oncogenic pathways [66, 67]. In such cases combinatorial regimen helps to overcome such problems [68].

## 3. Antibody-conjugated Nanoparticles for targeted Cancer Therapy

Recently research in nanoparticle based targeted therapy has gained momentum which saw the use of a full spectrum of nanoparticles in diagnostic and therapeutic applications of cancer [69]. Antibody-NP conjugates are being used for targeted delivery of chemotherapeutics and are considered as better therapeutic agents compared to NP conjugates due to their ability to circumvent some of the problems associated with direct conjugates, such as possible inactivation of the drug and the release of the drug in nonspecific areas once internalized into endosomal/ lysosomal vesicles through pH labile or reducible linkers [70, 71]. Moreover, the limitation with respect to the amount of drug that can be delivered to targeted area with direct antibody conjugated drug can be overcome by the use of antibody-NP complexes, which maximize the concentration of drug that can be targeted to the disease site. Recent studies focused on the development of antibody-coated lipid and non-lipid based nanoparticulates for antitumor research. The nanoparticulate antibodytargeting research is focused on antitumor strategies, where the antibody is used to target cell-surface markers of disease which are frequently upregulated or are specifically expressed in tumor cells [70-72].

Thus, the cancer drug discovery involves genome wide sequencing, understanding molecular pathology through bioinformatics and systems biology approaches for understanding how cancer cells can be targeted through single agents or on several fronts through drug combinations [73-75]. Although targeted treatment is considered a breakthrough in cancer treatment, latest research findings show that tumor heterogeneity with respect to molecular targets cause failure in many cases. This lead to evolution of concept of matching a patient to treatment which in other words is known as personalized medicine. In order to find the most effective treatment, the patient will be screened for the genes, proteins, and other factors unique to your tumor. After identifying the appropriate molecular targets the best suitable treatment will be recommended. Personalized medicine is gaining importance which ensures that the right drug is given to the right patient at the right time whereby maximum therapeutic benefit to patients is achieved. Pharmacologists and basic researchers are working together towards the discovery of effective and safe clinical candidates against the new targets trying to bridge the gap frequently referred to as technically druggable.
